# The long non-coding RNA *PCGEM1* is regulated by androgen receptor activity *in vivo*

**DOI:** 10.1186/s12943-015-0314-4

**Published:** 2015-02-21

**Authors:** Abhijit Parolia, Francesco Crea, Hui Xue, Yuwei Wang, Fan Mo, Varune Rohan Ramnarine, Hui Hsuan Liu, Dong Lin, Nur Ridzwan Nur Saidy, Pier-Luc Clermont, Hongwei Cheng, Colin Collins, Yuzhuo Wang, Cheryl D Helgason

**Affiliations:** Honours Biotechnology Program, Department of Microbiology and Immunology, University of British Columbia, Vancouver, BC Canada; Experimental Therapeutics, BC Cancer Research Centre, 675 West 10th Avenue, Vancouver, BC V5Z 1L3 Canada; The Vancouver Prostate Centre, Vancouver General Hospital, Vancouver, BC Canada; Interdisciplinary Oncology Program, Faculty of Medicine, University of British Columbia, Vancouver, BC Canada; Department of Urologic Sciences, University of British Columbia, Vancouver, BC Canada; Department of Surgery, University of British Columbia, Vancouver, BC Canada

**Keywords:** Long non-coding RNAs, lncRNAs, *PCGEM1*, *PRNCR1*, *PCAT18*, Androgen receptor, AR regulation, Sub-cellular localization, Prostate cancer

## Abstract

**Background:**

Long non-coding RNAs (lncRNAs) can orchestrate oncogenic or tumor-suppressive functions in cancer biology. Accordingly, *PCGEM1* and *PRNCR1* were implicated in progression of prostate cancer (PCa) as transcriptional co-regulators of the androgen receptor (AR). However, these findings were recently refuted asserting that neither gene physically binds to the AR. Despite evidence for differing AR transcriptional programs *in vivo* and *in vitro*, studies investigating AR-regulation of these genes hitherto have only been conducted *in vitro*. Here, we further examine the relevance of *PCGEM1* and *PRNCR1* in PCa, and their relationship with AR signaling, using patient-derived xenograft models.

**Findings:**

RNA sequencing of two distinct androgen-dependent models shows *PCGEM1* to be considerably expressed, while *PRNCR1* showed scant basal expression. *PCGEM1* was sharply down-regulated following castration and up-regulated upon AR activation *in vivo*. However, we found no parallel evidence following AR stimulation *in vitro*. A *PCGEM1*-associated gene expression signature (PES) was significantly repressed in response to androgen ablation therapy and in hormone-refractory versus hormone-naïve PCa patients. Furthermore, we found *PCGEM1* was uniformly distributed in PCa cell nucleus and cytoplasm which remained unaltered upon AR transcriptional activation. *PCGEM1* was up-regulated in primary PCa but not in metastasized PCa. Accordingly, the PES was significantly down-regulated in advanced and higher grade PCa patients from multiple independent studies.

**Conclusion:**

Our results demonstrate *PCGEM1* as an *in vivo* androgen-regulated transcript with potential nuclear and/or cytoplasmic function(s). Importantly, the clinical expression profile of *PCGEM1* implicates it in the early stages of PCa warranting further research in this direction.

**Electronic supplementary material:**

The online version of this article (doi:10.1186/s12943-015-0314-4) contains supplementary material, which is available to authorized users.

## Introduction

In recent years, long non-coding RNAs (lncRNAs) have emerged as major contributors to cellular homeostasis as well as initiation and progression of numerous diseases [1], including prostate cancer (PCa) [2]. The latest GENCODE v7 project annotated 14,880 human lncRNA transcripts with only a few characterized to date [3]. Of the lncRNAs functionally validated in various human malignancies, a majority have been identified as constituents of oncogenic or tumor suppressive pathways [4,5]. Some prominent lncRNAs implicated in prostate carcinogenesis and its progression include prostate cancer associated transcript 1 (*PCAT1*) [6], second chromosome locus associated with prostate 1 (*SChLAP1*) [7], prostate cancer associated 3 (*PCA3*) [8], prostate cancer gene expression marker 1 (*PCGEM1*; aka *PCAT9*) [9] and prostate cancer associated non-coding RNA 1 (*PRNCR1*; aka *PCAT8*) [10]. Notably, *SChLAP1* has been extensively validated in the clinics as a biomarker of aggressive PCa [11] and PCA3 is currently used in diagnostic tests [12]. Recently, we described *PCAT18* (aka *Loc728606*, *Linc01092*) as a mediator of metastatic progression based on expression profiling of our patient-derived PCa xenograft models from the Living Tumor Laboratory (LTL) [13]. *PCGEM1*, a highly prostate-specific transcript, was one of the first oncogenic lncRNAs to be described in PCa [9]. Subsequently, its over-expression was reported to attenuate the apoptotic response [14] and also promote cell proliferation and colony formation [15]. On the other hand, *PRNCR1* is not as well investigated, although its knockdown reportedly inhibits cell viability [10]. Recently, both of these lncRNAs occupied center stage due to their labeling as androgen receptor (AR)-interacting genes [16] – a claim now disputed [17].

AR is a ligand-responsive regulatory protein that mediates the effector functions of androgenic hormones in PCa. It is well established that sustained AR activity is indispensable for PCa cell survival and disease progression, even following androgen-deprivation therapy [18-20]. This “AR addiction” has led to many studies investigating genes serving as conduits for aberrant restoration of AR activity in recurrent tumors as potential therapeutic targets. In this regard, Srikantan et al. described *PCGEM1* as an AR regulated gene. Later on, *PCGEM1* acting in complicity with *PRNCR1* was shown to physically bind to the AR, thereby facilitating its ligand-independent transcriptional activity in castration resistant PCa (CRPC) [16]. In contrast, a recent publication indicated that neither *PCGEM1* nor *PRNCR1* interacted with the AR to render androgen-independence, and that both genes had no prognostic relevance in PCa [17]. Furthermore, the latter study found no evidence of *PCGEM1* and *PRNCR1* transcripts being AR regulated.

Notably, all published data on the relationship between AR and *PCGEM1/PRNCR1* hitherto have been derived from *in vitro* experiments, using androgen-sensitive LNCaP cells [9,17]. There is substantial evidence that transcriptional regulation of genes by a transcription factor is highly dynamic and cellular context-specific [21]. In this light, AR was recently demonstrated to induce varied and distinct AR transcriptional programs in patient tumor tissue as opposed to PCa cell lines [22]. This puts in doubt the suitability of cell line models for studying the transcriptional activity of the AR. In view of this, we set out to specifically investigate whether *PCGEM1* and *PRNCR1* are relevant in PCa and/or regulated by AR using our LTL patient-derived xenograft PCa models. The LTL has established a large panel of patient-derived PCa xenograft models that, unlike cell lines, retain key biological properties of the original malignancy, including histopathology, genomic profile, cellular heterogeneity, and invasive and metastatic ability [23].

## Findings and discussion

As a first step, we profiled our two AR+/androgen dependent PCa xenograft models – LTL-331 and LTL-313B – for expression of both lncRNAs using RNA Sequencing. While *PCGEM1* was considerably expressed (>500 FPKM; fragments per kilobase of exon per million fragments mapped), the expression of *PRNCR1* was <8 FPKM in both models (Additional file 1: Table S1). Such scant expression of *PRNCR1* raises serious questions about its biological relevance in AR-dependent PCa. Notably, this negligible expression of *PRNCR1* is in accordance with a more extensive clinical dataset that was recently published [17]. Together, these data weaken the claim made by Yang et al. [16] that *PRNCR1* plays a vital role in directing the transcriptional activity of AR. We, therefore, focused solely on *PCGEM1* for the remainder of our study.

### AR regulates expression of *PCGEM1 in vivo*

**Figure 1:**
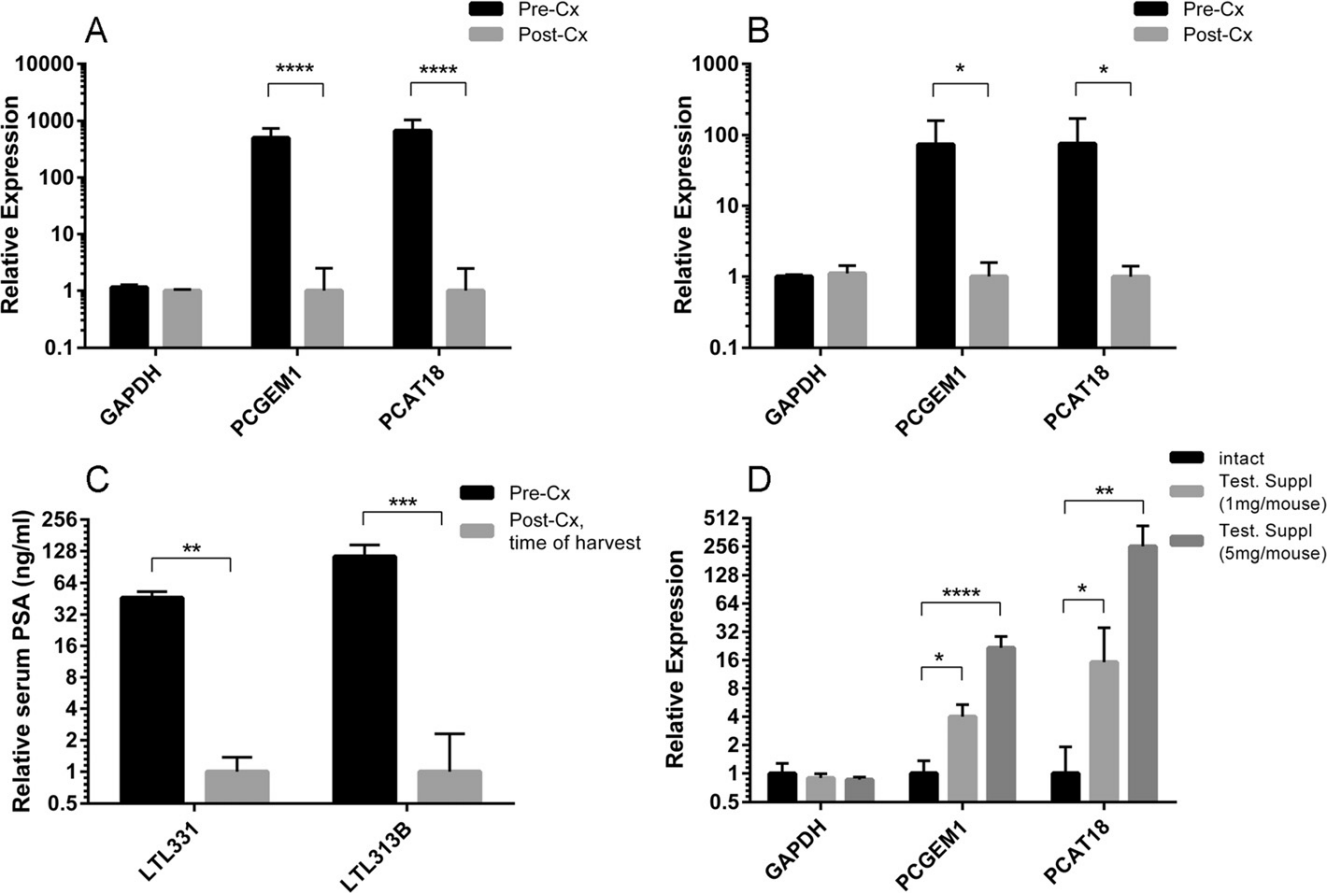
**Expression of** ***PCGEM1*** **in response to AR manipulation** ***in vivo*** **. A**, Expression (qPCR) of the labeled genes in LTL-331 tumor line from intact mice supplemented with testosterone (Test.) (n = 3; 5.0 mg/mouse) and 3 weeks after surgical castration (n = 4). Expression of all genes is referenced to average expression levels of HPRT1 and GAPDH and is expressed relative to the gene’s expression under castrate conditions. ****p < 0.0001 (unpaired, 2-tailed *T* test). **B,** Expression of the labeled genes (qPCR) in LTL-313B tumor line pre-castration (n = 3) and 12 weeks after castration (n = 3). Expression data is referenced and represented as described in the legend of Figure 1A. *p < 0.05 (unpaired, 2-tailed *T* test). **C**, Serum PSA levels in corresponding animals bearing LTL-331 or LTL-313B xenograft in A and B, respectively, just prior to castration and at the time of tissue collection after castration. Data is depicted relative to serum PSA values at post-castration time-points. **p < 0.01, ***p < 0.001 (unpaired, 2-tailed *T* test) **D**, Expression levels (qPCR) of the labeled genes in intact mouse hosts (n = 3) or intact hosts supplemented with two distinct dosages of pure testosterone (Test.) (1.0 mg/mouse or 5.0 mg/mouse, n = 4 and n = 3 respectively). Expression data is referenced as in Figure 1A and is depicted relative to the gene’s expression in intact mice. *p < 0.05, **p < 0.01, ****p < 0.0001 (2-Way ANOVA and Tukey’s post-test). For all the sub-figures, columns represent mean expression value for the biological replicates (each gene is quantified in triplicates), and bars represent standard deviation. TaqMan assay IDs for all the genes are listed in “Additional file 1: Table S4”.

We performed surgical castration (androgen ablation) of mice bearing the LTL-331 PCa xenograft. Tissue samples were collected just prior to castration and 3 weeks after castration, and were analyzed for gene expression. We observed a significant >500-fold decrease in *PCGEM1* expression post-castration relative to the pre-castration level (Figure 1A). Notably, this down-regulation was persistent (>180-fold) even twelve weeks following castration (Additional file 2: Figure S1). In agreement with this data, qPCR analysis on RNA collected before and 12 weeks after castration from a second *in vivo* model, LTL-313B, confirmed the down-regulation of *PCGEM1* (>70-fold; Figure 1B). In both models, reduced AR activity in response to castration was confirmed by the comparable down-regulation of *PCAT18* (Figure 1A,B), an AR-regulated lncRNA [13], and reduction in serum prostate-specific antigen (PSA) levels (at least >45-fold; Figure 1C). These findings led us to investigate the response of *PCGEM1* to AR stimulation *in vivo*. This was achieved by using intact mouse hosts supplemented with or without pure testosterone at two distinct dosages (1.0 mg and 5.0 mg/mouse) for PCa xenotransplantation of the LTL-331 tumor line. Corresponding to the dosages, serum testosterone levels of 3.96 ± 0.679 ng/ml and 20.6 ± 14.1 ng/ml were achieved in the testosterone supplemented animals versus 1.95 ± 0.106 ng/ml in the intact hosts. Notably, the hosts supplemented with pure testosterone showed a dose-dependent up-regulation of *PCGEM1*, from 5-fold to greater than 22-fold, relative to the hosts without the augmented testosterone (Figure 1D). This coincided with a comparable *PCAT18* up-regulation and serum PSA jump of >2-fold at both dosages.

**Figure 2:**
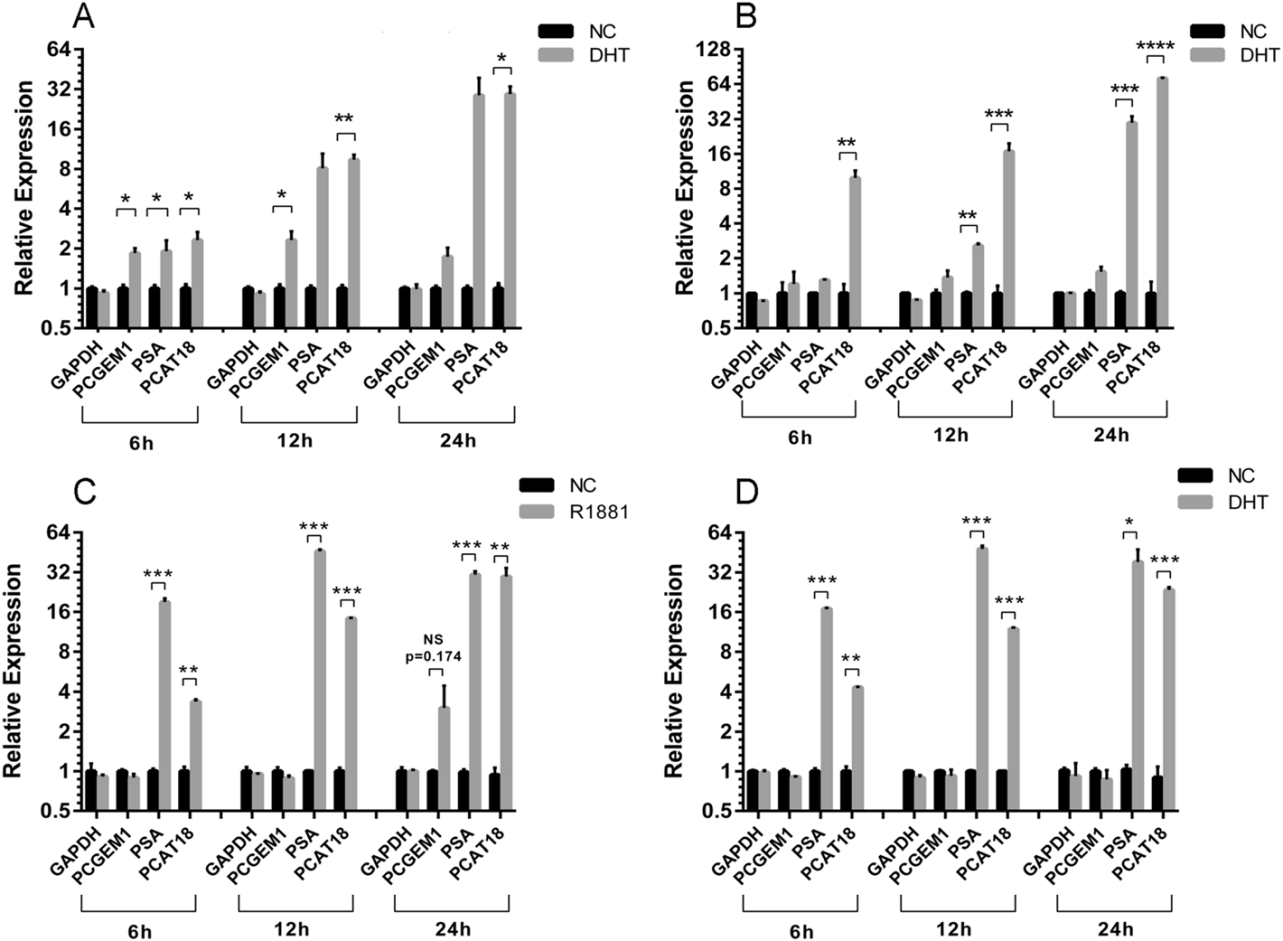
**Expression of** ***PCGEM1*** **in response to AR activation** ***in vitro*** **.** Expression levels (qPCR) of the labeled genes in **A**, LNCaP cells and **B**, VCaP cells treated with DHT at 10nM for 6 h, 12 h and 24 h. **C**, Expression (qPCR) of the labeled genes in LNCaP cells at 6 h, 12 h and 24 h after treatment with R1881 (10nM) or **D**, with a super-physiological dosage of DHT (100nM). For all the sub-figures, the columns represent mean expression value (2 independent experiments with each gene quantified in triplicates), bars represent standard deviation. NS, not significant; *p < 0.05; **p < 0.01; ***p < 0.001; ****p < 0.0001 (unpaired, 2-tailed *T* test).

Notably, previous *in vitro* studies investigating AR-regulation of *PCGEM1* produced conflicting results. Srikantan et al. [9] concluded *PCGEM1* is AR-regulated, while Prensner et al. [17] found no up-regulation of *PCGEM1* upon AR stimulation. The reason for this discrepancy could lie in the varied experimental conditions employed; in particular, the use of different agonists (viz. dihydrotestosterone (DHT) and synthetic R1881) for AR activation. Although the patterns of DHT-mediated and R1881-mediated gene expression changes are largely parallel, they do not completely overlap [24]. Consequently, we repeated this experiment using DHT, a physiological AR ligand, coupled with the sensitive TaqMan qPCR for gene quantification. Two AR+ PCa cell line models that expressed the highest levels of *PCGEM1*, LNCaP and VCaP, were chosen for the *in vitro* studies (Additional file 2: Figure S2). DHT treatment (at 6 h and 12 h) induced a modest (1.8 – 2.2-fold) up-regulation of *PCGEM1* in LNCaP cells (Figure 2A). However, the activation of *PCGEM1* did not continue to escalate in a manner similar to that of canonical AR-regulated genes such as PSA and *PCAT18* over extended time-points (12 h and 24 h). Similar observations were made in VCaP cells upon treatment with DHT (10nM; Figure 2B). Most remarkably, the weak AR-activation of *PCGEM1 in vitro* is in accordance with the recent observation that genes actively regulated by the AR in patient tumor tissue show a strong *in vivo* response to castration but a distinctly lesser response to androgen stimulation *in vitro* [22]. Notably, also contradicting the original findings [9], no significant increase in *PCGEM1* expression was observed *in vitro* (at 6 h, 12 h or 24 h) following stimulation of LNCaP cells with R1881 – a synthetic AR ligand (at 10nM; Figure 2C). Similar results were obtained on treatment with a super-physiological concentration of DHT (100nM; Figure 2D). Taken together, our results categorically demonstrate that substantial regulation of *PCGEM1* expression by androgen occurs exclusively *in vivo*.

### AR activation does not alter the uniform sub-cellular localization of *PCGEM1*

It was previously suggested that a direct AR-*PCGEM1* interaction regulates AR’s transcriptional activity [16]. In line with this mechanistic model, *PCGEM1*’s suspected function as a transcriptional co-regulator would imply it to be predominantly contained in the nucleus, in particular when the AR is transcriptionally active. However, sub-cellular localization of *PCGEM1* had never been investigated. Addressing this issue, we performed cellular fractionation of PCa patient-derived xenograft cells to isolate the nuclear and cytoplasmic RNA fractions, and quantified gene expression.

**Figure 3:**
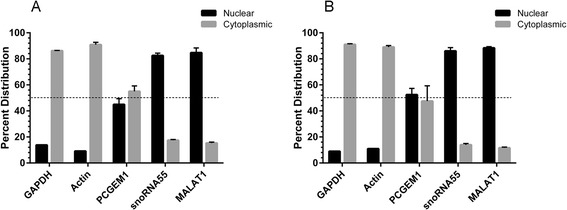
**Subcellular localization of** ***PCGEM1*** **with or without AR activation** ***in vivo*** **.** Sub-cellular localization of *PCGEM1*, GAPDH mRNA, Actin mRNA, *snoRNA55* and *MALAT1* in **A**, LTL-331 cells harvested from intact mouse hosts (n = 2) and **B**, intact hosts supplemented with pure testosterone (Test. Suppl; 5.0 mg/mouse, n = 2). For each gene, expression in nuclear and cytoplasmic fractions is represented as a percentage of the total expression. The dashed line indicates the 50% distribution mark. The columns represent mean % distribution value for the biological replicates (each gene is quantified in triplicates), and the bars represent standard deviation.

Our data shows that *PCGEM1* is evenly distributed between the nucleus and the cytoplasm of LTL-331 cells harvested from intact mouse hosts or intact hosts supplemented with testosterone (Figure 3A,B). Expected cytoplasmic localization of GAPDH and Actin (>85% for both) and nuclear localization of small nucleolar RNA 55 (*snoRNA55*) and *MALAT1* (>80% for both) authenticate our findings. A similar sub-cellular distribution of *PCGEM1* was observed in DHT treated and untreated LNCaP cells as well (Additional file 2: Figure S3A, B). While these results do not conclusively rule out the possibility that *PCGEM1* may be interacting with the AR, they demonstrate for the first time that *PCGEM1* is not selectively localized in the nucleus; or is shuttled to it upon transcriptional activation of the AR. These findings also highlight the gap in our understanding of lncRNAs with proposed cell compartment-specific functions. The unresolved question is whether lncRNAs with nuclear roles are contained in the nucleus throughout their lifetime, or do they also shuttle to the cytoplasm where they can adopt additional roles? For instance, *H19*, one of the most well characterized lncRNAs, is associated with both nuclear and cytoplasmic functions i.e. chromatin modulation [25] and micro-RNA generation [26,27], respectively. Collectively, our results warrant further verification of the alleged function of *PCGEM1* as a transcriptional co-regulator of the AR, in addition to investigating its potential role in the cytoplasm. For detailed information about all the experiments refer to “Additional file 3: Supplementary methods and materials.”

### *PCGEM1* is implicated in early stages of PCa

Our results to this point indicate *PCGEM1* to be an *in vivo* androgen regulated gene, with uniform distribution in PCa cell nucleus and cytoplasm. One remaining question, however, is the stage of PCa in which *PCGEM1* might be biologically most relevant. To this end we investigated a patient database consisting of microarray and clinical data on 131 primary PCa, 19 metastasized PCa and 29 normal prostatic tissue samples [28]. Here we observed that *PCGEM1* was significantly up-regulated only in primary PCa but not in metastasized PCa relative to normal prostate tissue. In fact, *PCGEM1* expression was significantly down-regulated in metastasized tumors relative to primary tumors (Additional file 2: Figure S4). Adding to a recent study discrediting the involvement of *PCGEM1* in progression of localized PCa to CRPC [17], our analysis further raises the question of the biological relevance of *PCGEM1* in survival and proliferation of metastasized PCa cells themselves. In view of this, *PCGEM1* is most likely implicated only in the early stages of the disease warranting further experimental validation. To explore this further, we performed Pearson’s Correlation analysis to identify genes that are positively associated with *PCGEM1* expression, hereafter referred to as *PCGEM1*-associated gene expression signature (PES) (Additional file 1: Table S2), and uploaded the list into the Oncomine database. As previously demonstrated for *PCGEM1*, the PES was also significantly up-regulated in PCa relative to other neoplasms and in PCa versus normal prostate tissue (Table 1). Corroborating the relevance of *PCGEM1* in early stages of PCa, PES was consistently repressed in metastatic and high Gleason PCa relative to primary and low Gleason neoplasms, respectively (Table 1). Notably, this trend was observed in several independent patient cohorts totaling more than 1100 samples. Accordingly, the PES was significantly repressed in patients exhibiting poor clinical outcomes.

**Table 1:** ***PCGEM1*** **-associated expression signature in PCa patient samples**

**Concept**	**Studies (Up/Down)**	**P-value**	**Odds ratio**	**Total samples**
PCa vs. Other Neoplasms	1/0	4.44E-15	30.9	1468
PCa vs. Normal Prostate	5/0	1.88E-10 - 2.00E-03	14.5 - 4.5	299
Metastatic PCa vs. Primary PCa	0/6	6.69E-13 - 5.00E-03	27.0 - 4.5	448
High Gleason PCa vs. Low Gleason PCa	0/5	8.01E-11 - 6.00E-3	16.8 - 5.0	676
Hormone-Refractory PCa vs. Hormone-naïve PCa	0/1	4.74E-05	9.6	20
Clinical Outcomes				
Reoccurrence at 3 or 5 years	0/2	1.45E-06 - 4.52E-04	8.4 - 5.1	168
Dead at 3 or 5 years	0/2	4.65E-07 - 9.02E-04	20.6 -7.8	721

The MSKCC database was queried using cBioPortal for genes that are co-expressed with *PCGEM1* (Pearson’s Correlation > 0.50), which were then uploaded into the Oncomine database (thresholds: p-value < 0.01, odds ratio > 2). Only clinically-related Oncomine concepts were analyzed and are listed in the first column. Here, *PCGEM1* expression in PCa was compared to 15 other neoplasms. The second column summarizes the total number of independent studies showing either up- or down-regulation of PES for the corresponding concept. “Total Samples” reflect the grand total of number of patients in every study that met the threshold for the corresponding concept. Oncomine (Compendia Bioscience) was used for statistical analysis.

A review of literature also reveals that *PCGEM1* is up-regulated in normal prostate epithelial cells of men with a family history of PCa [15] and certain single-nucleotide polymorphisms in the *PCGEM1* gene are associated with a higher risk of PCa development in Chinese men [29]. Together, these findings support the testable hypothesis that *PCGEM1* is more important in the early stages, possibly initiation, of primary PCa. Interestingly, the *PCGEM1* signature was also under-expressed in hormone-refractory PCa relative to hormone-naïve PCa (Table 1); and the pathway analysis revealed PES to be most significantly down-regulated (p = 5.47E-14, odds ratio = 121.4) in patients receiving androgen-deprivation therapy (Additional file 1: Table S3) lending further support to *in vivo* AR-regulation of *PCGEM1*.

Our work brings in light an even more pertinent concern with exploring AR transcriptional activity using cell line models. Recently, cell line models in accordance with their metastatic origin were demonstrated to exhibit the AR regulatory program that is active in metastatic advanced PCa as opposed to primary PCa [22]. The AR regulatory activity in cultured cell line models (including LNCaP) had a greater overlap to CRPC (31% overlap) than untreated primary PCa (merely 3% overlap) (see [22]). Besides the absent cell microenvironment, this describes further limitations of cell line models for investigating AR-regulation, in particular for genes with a clinical expression profile akin to that of *PCGEM1*. Simultaneously, it underscores the importance of using clinically relevant models that best represent the original malignancy. With mounting evidence for lncRNAs implicated in carcinogenesis and cancer progression, we regard lncRNAs as essential in decrypting cancer cell biology. Our study corroborates the irrelevance of *PRNCR1* in PCa, and confirms *PCGEM1* as an *in vivo* AR-regulated transcript as well as rationalizes its oncogenic involvement in early stages of the disease.

## Additional files

Additional file 1: Table S1. RNA-Sequencing data for *PCGEM1* and *PRNCR1* in LTL xenograft models. Transcript name, ensembl ID, transcript class, chromosomal coordinates and FPKM values in LTL-331 and LTL-313B for GAPDH (control), *PCGEM1* and *PRNCR1*. **Table S2.** *PCGEM1*-associated expression signature (PES) in PCa Samples. *PCGEM1* was queried using cBioPortal in the prostate adenocarcinoma (MSKCC, Cancer Cell 2010) cancer study for mRNA expression data. List of genes with the highest expression correlation with *PCGEM1* (pearson’s correlation > 0.50) was obtained from the “Co-Expression” module on cBioPortal. A total of 29 genes, listed in the first column, were significantly co-expressed with *PCGEM1*. Second and third columns list the Pearson’s and Spearman’s correlation values that were obtained from the cBioPortal analysis. **Table S3.** Literature-defined concepts associated with PES. All transcripts that positively associated with *PCGEM1* (see Additional file 1: Table S2) were uploaded into the Oncomine database and analyzed for “literature-defined concepts” (thresholds: P-value < 0.01. odds ratio > 2). Here, we show only the top 5 solid tumor-related concepts significantly associated with PES. **Table S4.** Gene expression TaqMan assays. All assays were purchased from Life Technologies and were pre-designed for the probe to span exons except for *snoRNA55*, for which both the primers and probe map within a single exon. All probes had the FAM reporter signal. Additional file 2: Figure S1. Expression of *PCGEM1* in LTL-331 castration time series. A, Expression (qPCR) of the labeled genes in LTL-331 tumor line from mice supplemented with testosterone (Test.) (n = 3, 5.0 mg/mouse), or after surgical castration (n = 1, at 1, 2, 3, 8 and 12 weeks). Expression of all genes is referenced to average expression levels of HPRT1 and GAPDH and is expressed relative to the gene’s expression in testosterone-supplemented mice. Dots represent mean expression value and bars represent standard deviation. B, Serum PSA levels from the corresponding LTL-331 xenograft bearing animals in A. **Figure S2.** *PCGEM1* basal expression profile in a panel of PCa cell lines. Expression levels of *PCGEM1* in a non-neoplastic prostate cell line and a panel of commonly used AR+ and AR- PCa cell line models. *PCGEM1* expression is referenced to average expression levels of HPRT1 and GAPDH and is expressed relative to its expression in 22RV1 cells where it is barely expressed (Ct value = ~41). No column represents that *PCGEM1* transcript was undetectable in the corresponding cell line using the Ct cutoff of 45 cycles. Columns represent mean expression value and bars represent standard deviation. **Figure S3.** Sub-cellular localization of *PCGEM1* with or without DHT treatment *in vitro. E*xpression of the labelled genes (qPCR) in sub-cellular fractions of A, untreated LNCaP cells and B, LNCaP cells stimulated with DHT (10nM) for 12 h. Columns represent mean distribution value from 2 independent experiments and bars represent standard deviation. **Figure S4.** Taylor PCa cohort analysis. *PCGEM1* expression (microarray data) in 131 primary PCa, 19 partially paired secondary metastatic PCa tissues and 29 normal prostatic tissues. These are median-centered values where bars represent maximum and minimum value per group. NS, not significant; ***p < 0.001 (2-Way ANOVA and Tukey’s post-test). Additional file 3:
**Supplementary methods and materials.**
