# Supplementary material for: The long non-coding RNA PCGEM1 is regulated by androgen receptor activity in vivo
Source: Mol Cancer. 2015 Feb 21;14:46. doi: 10.1186/s12943-015-0314-4 (PMC4342943; doi:10.1186/s12943-015-0314-4)
Supplement: Additional file 1: Table S1. — RNA-Sequencing data for PCGEM1 and PRNCR1 in LTL xenograft models. Transcript name, ensembl ID, transcript class, chromosomal coordinates and FPKM values in LTL-331 and LTL-313B for GAPDH (control), PCGEM1 and PRNCR1. Table S2. PCGEM1-associated expression signature (PES) in PCa Samples. PCGEM1 was queried using cBioPortal in the prostate adenocarcinoma (MSKCC, Cancer Cell 2010) cancer study for mRNA expression data. List of genes with the highest expression correlation with PCGEM1 (pearson’s correlation > 0.50) was obtained from the “Co-Expression” module on cBioPortal. A total of 29 genes, listed in the first column, were significantly co-expressed with PCGEM1. Second and third columns list the Pearson’s and Spearman’s correlation values that were obtained from the cBioPortal analysis. Table S3. Literature-defined concepts associated with PES. All transcripts that positively associated with PCGEM1 (see Additional file 1: Table S2) were uploaded into the Oncomine database and analyzed for “literature-defined concepts” (thresholds: P-value < 0.01. odds ratio > 2). Here, we show only the top 5 solid tumor-related concepts significantly associated with PES. Table S4. Gene expression TaqMan assays. All assays were purchased from Life Technologies and were pre-designed for the probe to span exons except for snoRNA55, for which both the primers and probe map within a single exon. All probes had the FAM reporter signal. [file 12943_2015_314_MOESM1_ESM.docx]

Additional file 1

Table S1

| **Gene Name** | **Ensembl ID** | **Category** | **Chromosome Coordinates** | **FPKM in LTL-331** | **FPKM in LTL-313B** |
| --- | --- | --- | --- | --- | --- |
| GAPDH | ENSG00000111640 | protein-coding | chr12:6643093-6647537 | 139969.2 | 157461.8 |
| *PCGEM1* | ENSG00000227418 | lncRNA | chr2:193614571-193641621 | 2141.106 | 513.2022 |
| *PRNCR1* | ENSG00000224722 | pseudogene | chr8:128098508-128099755 | 4.7461147 | 7.871199 |

Table S2

| **Correlated Genes** | **Pearson’s Correlation** | **Spearman’s correlation** |
| --- | --- | --- |
| TMEFF2 | 0.76 | 0.79 |
| CNTNAP2 | 0.61 | 0.56 |
| AFF3 | 0.58 | 0.56 |
| RLN1 | 0.58 | 0.58 |
| DLGAP1 | 0.58 | 0.56 |
| EHHADH | 0.57 | 0.5 |
| SLC13A3 | 0.57 | 0.55 |
| MTOR | 0.56 | 0.51 |
| ABAT | 0.55 | 0.54 |
| CAMK1 | 0.55 | 0.54 |
| FAM13C | 0.55 | 0.55 |
| GLB1L3 | 0.54 | 0.53 |
| TBC1D4 | 0.54 | 0.53 |
| NCAPD3 | 0.54 | 0.55 |
| ACAD8 | 0.54 | 0.51 |
| INPP4B | 0.53 | 0.51 |
| C2ORF72 | 0.53 | 0.49 |
| ACADL | 0.52 | 0.49 |
| TFF3 | 0.52 | 0.46 |
| RAB6C-AS1 | 0.52 | 0.48 |
| KCNH6 | 0.52 | 0.51 |
| LPAR3 | 0.52 | 0.52 |
| FAM3B | 0.52 | 0.51 |
| RLN2 | 0.51 | 0.53 |
| POTED | 0.51 | 0.52 |
| COL28A1 | 0.51 | 0.49 |
| SPON2 | 0.5 | 0.48 |
| POTEC | 0.5 | 0.53 |
| EFCAB12 | 0.5 | 0.49 |

Table S3

| **Literature-Defined Concepts** | **P-value** | **Odds Ratio** |
| --- | --- | --- |
| Down-regulated genes in PCa in response to androgen ablation therapy | 5.47E-14 | 121.2 |
| Down-regulated genes in PCa cells in response to Resveratrol | 1.46E-05 | 19.2 |
| Down-regulated genes in PCa cells in response to Methylseleninic Acid (MSA) | 6.84E-05 | 21.2 |
| Down-regulated genes in HeLa cells following Camptothecin treatment | 9.23E-05 | 12.9 |
| Up-regulated genes in PCa cells in response to synthetic androgen R1881 | 9.08E-04 | 17.7 |

Table S4

| **Gene Name** | **TaqMan Assay ID** |
| --- | --- |
| HPRT1 | Hs02800695_m1 |
| GAPDH | Hs02758991_g1 |
| *PCGEM1* | Hs01369007_m1 |
| *PCAT18* (aka *Loc728606*) | Hs03669364_m1 |
| PSA (aka KLK3) | Hs02576345_m1 |
| *snoRNA55* | Hs03298696_s1 |
| *MALAT1* | Hs00273907_s1 |
| Actin | Hs01060665_g1 |
