# Supplementary figures and images for: The long non-coding RNA PCGEM1 is regulated by androgen receptor activity in vivo
Source: Mol Cancer. 2015 Feb 21;14:46. doi: 10.1186/s12943-015-0314-4 (PMC4342943; doi:10.1186/s12943-015-0314-4)

Additional file 2

Figure S1


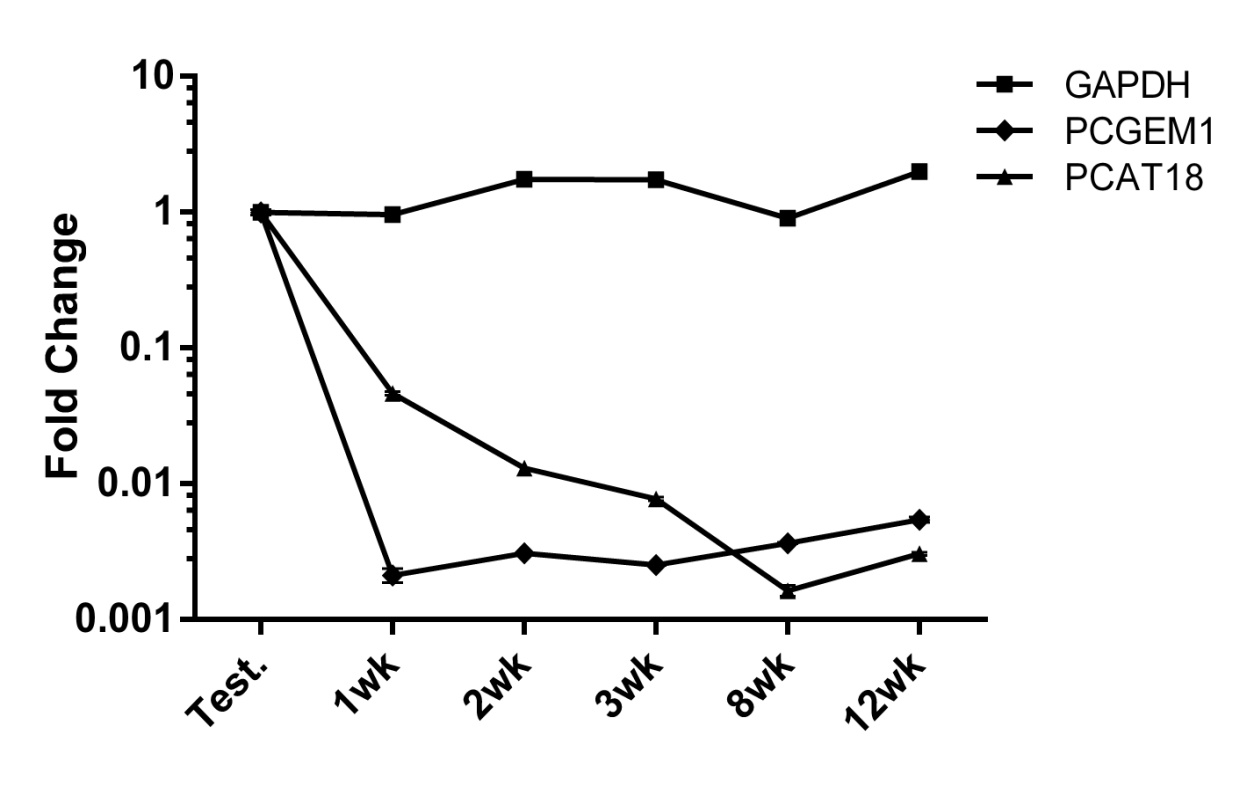

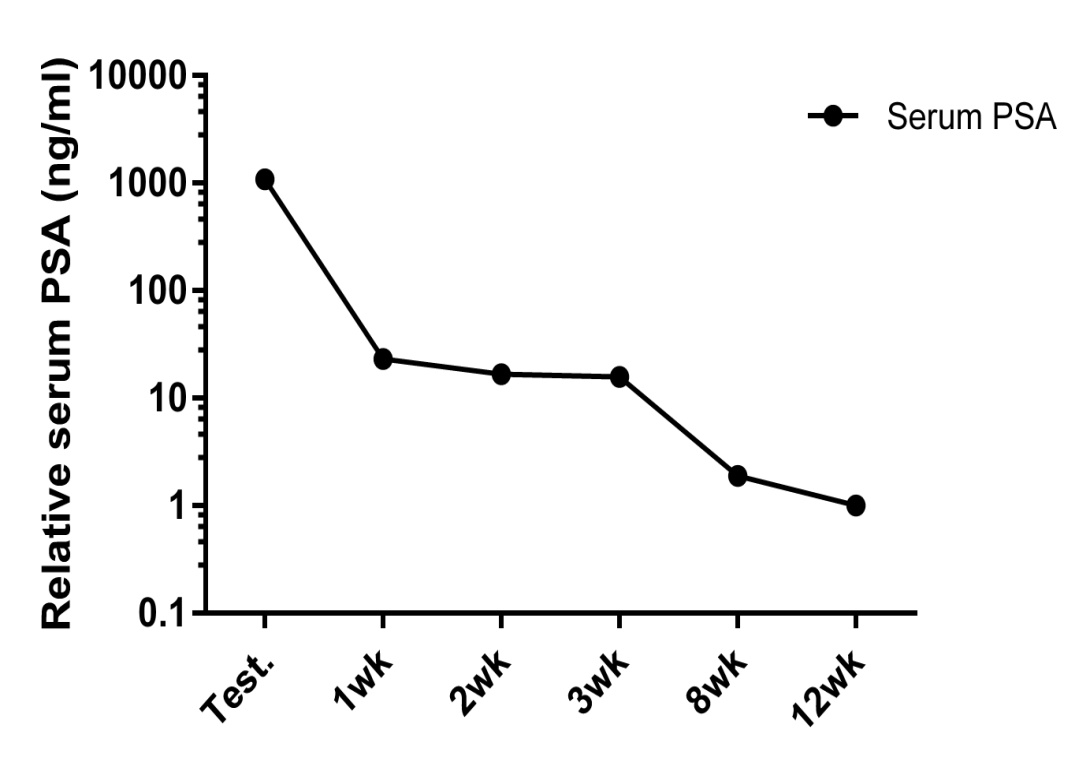


Figure S2


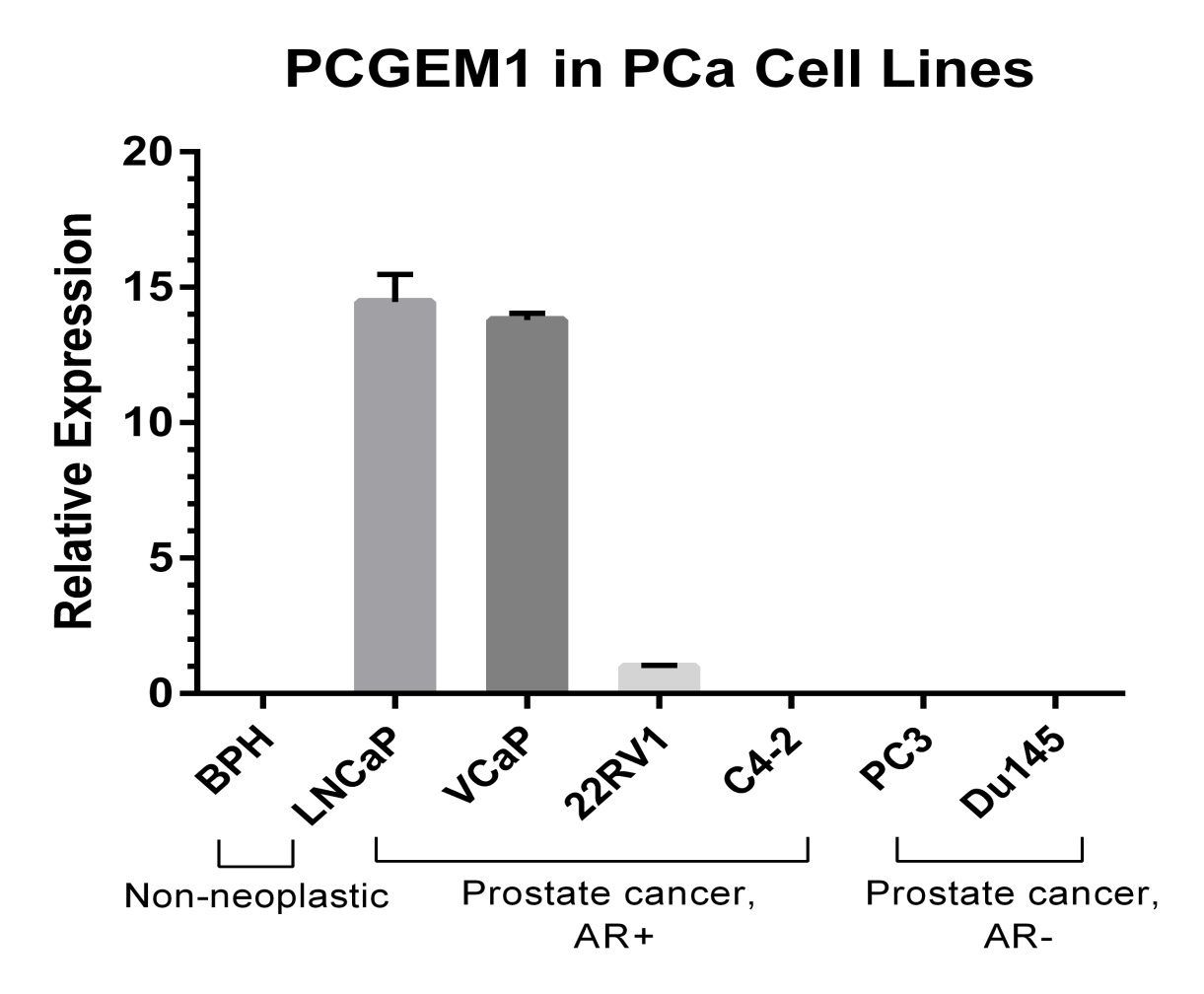


Figure S3

B

A


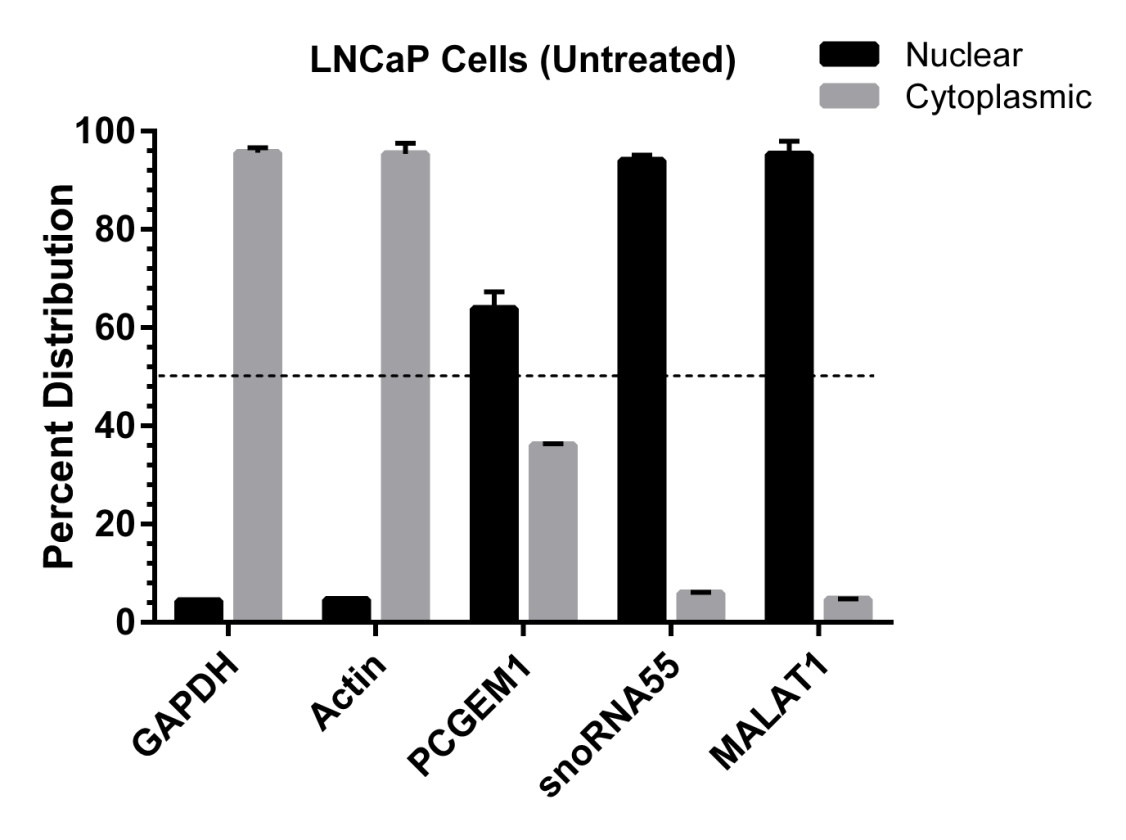

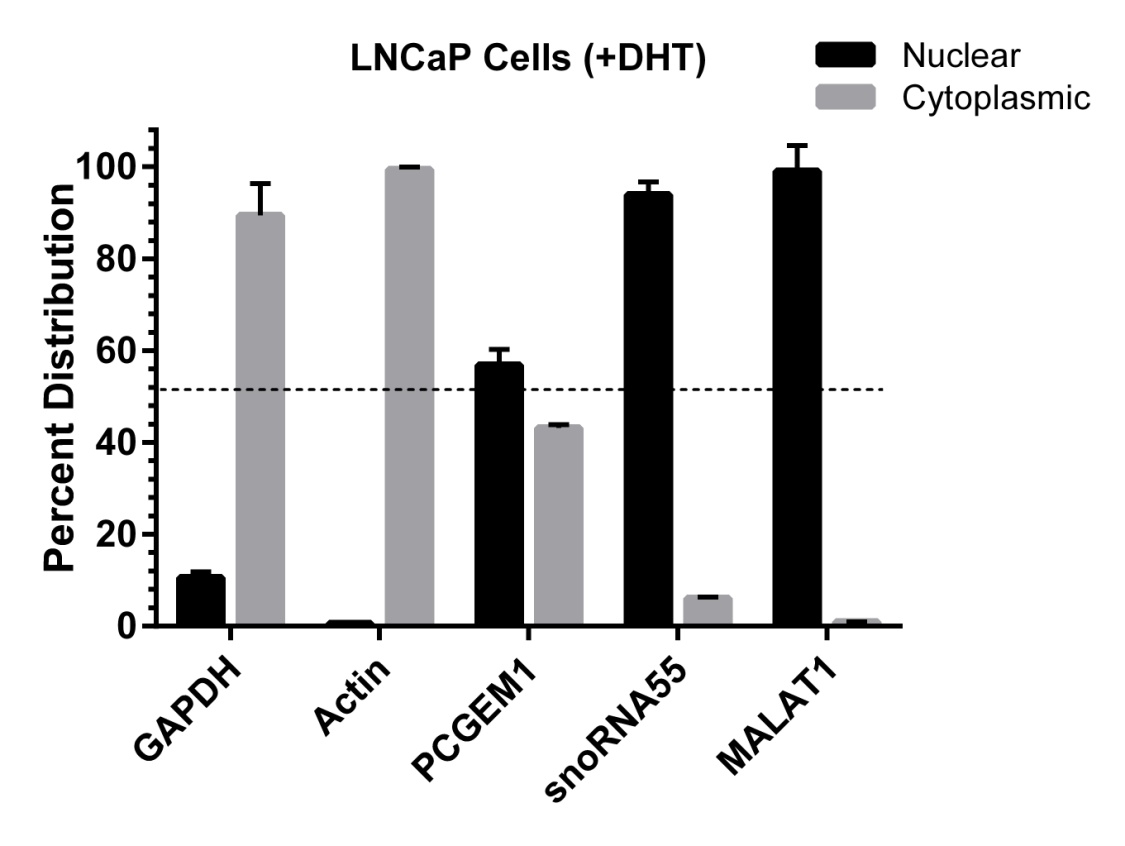


Figure S4


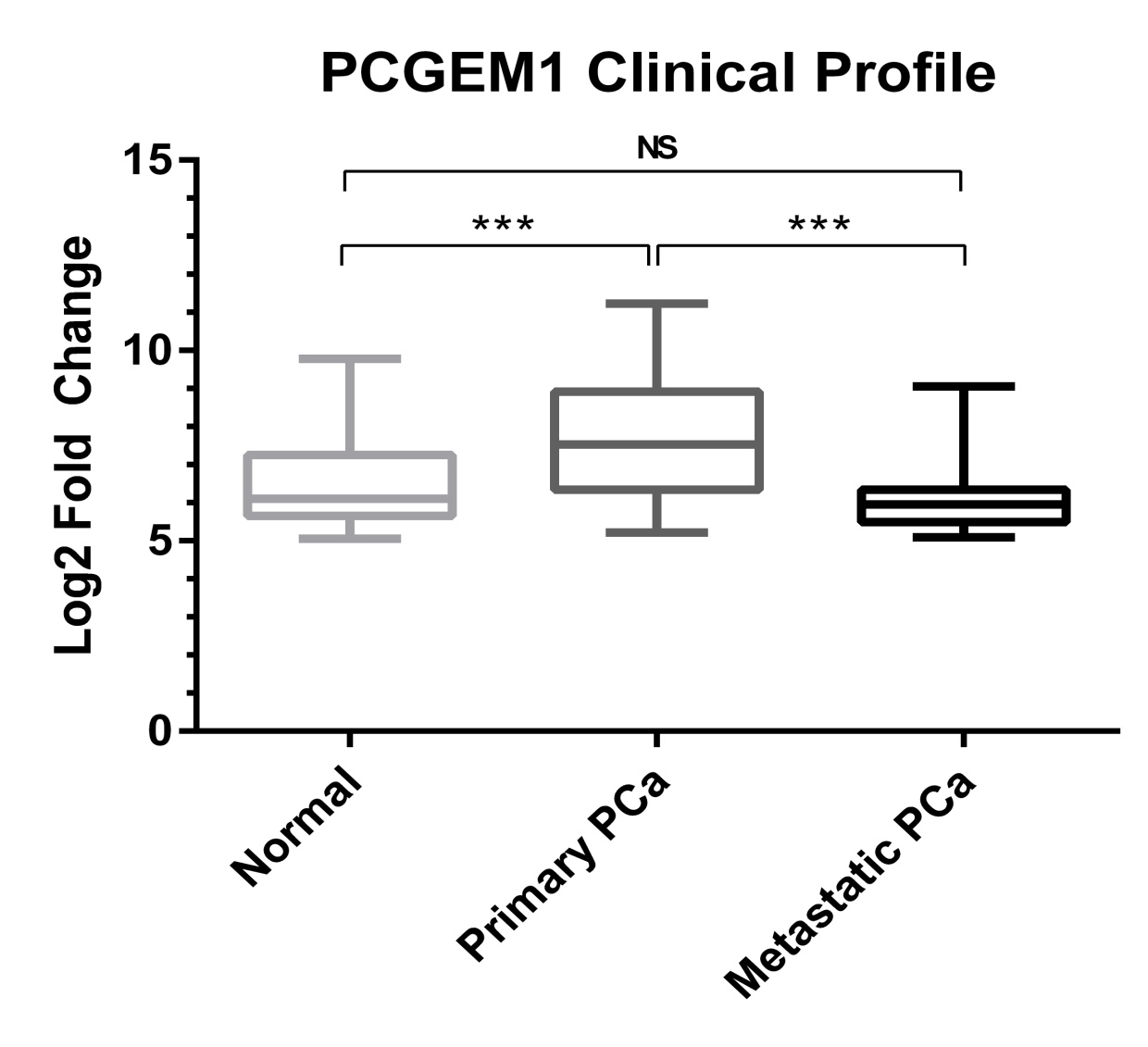

Supplement: Additional file 2: Figure S1. — Expression of PCGEM1 in LTL-331 castration time series. A, Expression (qPCR) of the labeled genes in LTL-331 tumor line from mice supplemented with testosterone (Test.) (n = 3, 5.0 mg/mouse), or after surgical castration (n = 1, at 1, 2, 3, 8 and 12 weeks). Expression of all genes is referenced to average expression levels of HPRT1 and GAPDH and is expressed relative to the gene’s expression in testosterone-supplemented mice. Dots represent mean expression value and bars represent standard deviation. B, Serum PSA levels from the corresponding LTL-331 xenograft bearing animals in A. Figure S2. PCGEM1 basal expression profile in a panel of PCa cell lines. Expression levels of PCGEM1 in a non-neoplastic prostate cell line and a panel of commonly used AR+ and AR- PCa cell line models. PCGEM1 expression is referenced to average expression levels of HPRT1 and GAPDH and is expressed relative to its expression in 22RV1 cells where it is barely expressed (Ct value = ~41). No column represents that PCGEM1 transcript was undetectable in the corresponding cell line using the Ct cutoff of 45 cycles. Columns represent mean expression value and bars represent standard deviation. Figure S3. Sub-cellular localization of PCGEM1 with or without DHT treatment in vitro. Expression of the labelled genes (qPCR) in sub-cellular fractions of A, untreated LNCaP cells and B, LNCaP cells stimulated with DHT (10nM) for 12 h. Columns represent mean distribution value from 2 independent experiments and bars represent standard deviation. Figure S4. Taylor PCa cohort analysis. PCGEM1 expression (microarray data) in 131 primary PCa, 19 partially paired secondary metastatic PCa tissues and 29 normal prostatic tissues. These are median-centered values where bars represent maximum and minimum value per group. NS, not significant; ***p < 0.001 (2-Way ANOVA and Tukey’s post-test). [file 12943_2015_314_MOESM2_ESM.docx]
