# Supplementary material for: The long non-coding RNA PCGEM1 is regulated by androgen receptor activity in vivo
Source: Mol Cancer. 2015 Feb 21;14:46. doi: 10.1186/s12943-015-0314-4 (PMC4342943; doi:10.1186/s12943-015-0314-4)
Supplement: Additional file 3: — Supplementary methods and materials. [file 12943_2015_314_MOESM3_ESM.docx]

# SUPPLEMENTARY METHODS AND MATERIALS

**Cell culture**

The human LNCaP and VCaP cell lines were purchased from the American Type Culture Collection. Unless otherwise specified, LNCaP cells were maintained in RPMI 1640 growth medium (Gibco) supplemented with 10% fetal bovine serum (FBS; Gibco). VCaP cells were cultured in ATCC-formulated DMEM growth medium supplemented with 10% FBS. Both cell lines were grown at 37⁰C in a humidified 5% CO_2_ incubator.

**Primary tissue digestion**

LTL-331 PCa tissue fragments were washed twice with ice-cold Hank’s Balanced Salt Solution – HBSS (Gibco), and then cut into smaller pieces (roughly 1mm^3^ is size) using sterile forceps and scalpels. Smaller fragments were then re-suspended in pre-warmed and filtered (0.2 µm) 1mg/ml Collagenase Type 1 (Gibco) solution in phosphate buffered saline (PBS). The mixture was incubated at 37°C for 1h with gentle stirring. The digested tissue mixture was next passed through a 70µm nylon cell strainer (BD Falcon) and the filtrate was collected into a 50mL centrifuge tube. Sieved cells were then washed once with HBSS solution via centrifugation (400g for 5mins). Subsequent to the wash, pelleted cells were used for sub-cellular RNA fractionation as described below.

**Patient-derived PCa xenograft**

Patient biopsy samples of primary prostate cancer (PCa) were collected at the BC Cancer Agency or the Vancouver Prostate Centre with the patient’s prior written consent. The University of British Columbia (UBC) - Research Ethics Board approved all surgical protocols involved in acquiring PCa biopsies from patients and their handling and use for research purposes (protocol #: H04-60131). The immune-compromised male NOD-SCID mice used for xenotransplantation were obtained from the British Columbia Cancer Research Centre - Animal Resource Centre (Vancouver, Canada). Protocols for all experimental work involving animals were approved by the Animal Care Committee at UBC (protocol #: A10-0100). PCa tumors were maintained via grafting in the sub-renal capsules as previously described [1], establishing first-of-its-kind xenograft PCa tumor lines. LTL-331 tumor line was derived from a single primary PCa biopsy. At the time of biopsy, the patient was diagnosed with Gleason Grade 9, pT3aN0M0 PCa adenocarcinoma. For LTL-313B, donor patient’s clinical information can be found in [2].

**RNA extraction and reverse-transcription**

Total RNA was extracted using the RNeasy Mini Kit (Qiagen) and reverse-transcribed using the QuantiTect Kit (Qiagen). Genomic DNA elimination is integrated in the reverse-transcription (cDNA synthesis) procedure and was carefully performed each time as per manufacturer’s instructions.

Sub-cellular RNA fractionation was performed using the Paris Kit (Ambion, Life Technologies) following the manufacturer’s instructions. Nuclear pellets were further purified by a secondary wash using the fractionation buffer, as per manufacturer’s instructions. RNA quality for both the nuclear and cytoplasmic fraction was assessed using the ND-1000 Spectrophotometer (NanoDrop Technologies). cDNA was synthesized using the QuantiTect Kit (Qiagen) as described above.

**RNA sequence analysis**

RNA sequenced reads were aligned to a customized reference sequence combining hg19 human and mm10 mouse genome using a splice aware aligner, STAR [4], for handling exonic reads and ones spanning exon-exon junctions. Ensembl release 75 was used for annotation of known transcript models. Mouse contaminated sequenced reads were identified and filtered out during the mapping stage. Based on sequence alignment, gene expression profiles for each LTL model were calculated based on the gene annotation (Ensembl release 75). Only reads which were unique to one gene and exactly corresponded to the gene exon-intron chain structure were assigned to the corresponding gene. Raw read counts were normalized by R package DESeq [5], which was designed for gene expression analysis of RNA-sequencing data.

**Quantitative real-time PCR**

All TaqMan assays for gene expression quantification were purchased from Applied Biosystems, Life Technologies. Assay IDs are listed in Additional file 1: Table S4. All assays were pre-designed for the primers and the probe to span exons except for *snoRNA55* for which both the primers and probe map within a single exon. Quantitative Real-time PCR (qPCR) was performed using cDNA, TaqMan Assay, and TaqMan Universal Master Mix II, with UNG (Applied Biosystems), on an ABI Prism 7900HT (Applied Biosystems) sequence detection system following manufacturer’s instructions. We employed the 2^-ΔΔCT^ method for calculating fold change values as described in [3]. Data from all samples were referenced to the average HPRT1 and GAPDH Ct values, and the fold change values were normalized (expressed relative) to the control samples in each experiment.

***In vivo* androgen deprivation**

Patient PCa tumor tissues were transplanted under the renal capsules of intact male NOD-SCID mice supplemented with testosterone pellets (5.0mg/mouse). For the LTL-331 tumor line, mice were euthanized and tumor tissues were harvested in their growth phase on reaching a volume of roughly 1cm^3^ (n=3), while others at this stage were surgically castrated and testosterone pellets were removed. Tumor tissues were collected at 3 weeks after castration and removal of the testosterone pellet (n=4). In addition to this, to generate an extensive LTL-331 castration time-series, tumor tissues were collected at 1, 2, 8 and 12 weeks time-points (n=1) after castration.

For the LTL-313B tumor line in mice (supplemented testosterone: 5.0mg/mouse), castration was performed when tumors were in their growth phase with a volume of roughly 1cm^3^ after transplantation. At this stage, pre-castration tissues were also collected (n=3). Post-castration tissues were harvested only at a single time point of 12 week post-castration (n=3). For all tissue samples, RNA was extracted and gene expression was quantified via qPCR as described above.

Serum PSA levels at the time of tumor tissue collection were quantified using the Total PSA kit (Roche Diagnostics) on a Cobas e411 analyzer (Roche Diagnostics), following manufacturer’s instructions.

***In vivo* androgen stimulation**

Patient PCa tumor tissues (LTL-331) were transplanted under the renal capsules of intact male NOD-SCID mice supplemented with (androgen stimulated) or without (control, n=3) a testosterone pellet at two distinct dosages (1.0mg/mouse, n=4 or 5.0mg/mouse, n=3). Mice were euthanized and tumor tissues were harvested in their growth phase when the tumor volume reached roughly 1cm^3^ in each case. Total RNA was extracted and gene expression was quantified as described above. For sub-cellular RNA fractionation, LTL-331 tissue was digested using the “primary tissue digestion” protocol described above (n=2 per group).

Serum PSA and testosterone levels at the time of tumor harvesting were quantified using the Total PSA and Testosterone II kit (Roche Diagnostics) on a Cobas e411 analyzer (Roche Diagnostics), following manufacturer’s instructions.

***In vitro* AR stimulation**

LNCaP or VCaP cells were seeded at 25% confluency in their corresponding complete growth medium 16h prior to the treatment to allow adherence to the culture plate surface. Complete medium was then aspirated and the cells were washed twice using phosphate buffered saline **(**PBS) solution. Cells were then placed in phenol-free RPMI 1640 (Gibco) + 10% Charcoal Stripped Serum (Gibco) growth medium supplemented with (treated) or without (negative control) physiological AR agonist dihydrotestosterone (DHT; Sigma Aldrich) at 10nM or 100nM for various durations, or with synthetic AR agonist R1881 (PerkinElmer) at 10nM for 6h, 12h and 24h. R1881 was dissolved in filter-sterilized ethanol and DHT was dissolved in filter-sterilized distilled water. Appropriate volume of the corresponding solvent was added to the negative control treatments.

At each time-point, cells were trypsinized and washed once with cold PBS. Subsequently, RNA was extracted and reverse-transcribed as described above and qPCR was performed to quantify gene expression.

**cBioPortal and oncomine analysis**

*PCGEM1* was queried in the Prostate Adenocarcinoma (MSKCC, Cancer Cell 2010) database for the mRNA expression using the cBioPortal (http://www.cbioportal.org/public-portal/). The *PCGEM1* associated transcripts (Pearson’s Correlation > 0.50) were obtained from the “Co-Expression” module on the cBioPortal website. These positively co-expressed genes were then uploaded into the Oncomine database (https://www.oncomine.com/resource/login.html) to investigate correlations with clinical variable and molecular pathways using the following thresholds: P-value<0.01 & Odds ratio>2.

**Statistical analyses**

Data is shown as mean values ± standard deviation for all experiments. All *in vitro* experiments were repeated at least twice. Data are graphed and analyzed using GraphPad Prism 6 software. Statistical tests performed in each case are detailed in the corresponding figure legend.

**SUPPLEMENTARY REFERENCES**

1. Lin D, Wyatt AW, Xue H, Wang Y, Dong X, Haegert A, et al. High fidelity patient-derived xenografts for accelerating prostate cancer discovery and drug development. [Cancer Res.](http://www.ncbi.nlm.nih.gov/pubmed/?term=24356420) 2014; 74(4):1272-1283.
2. [Crea F](http://www.ncbi.nlm.nih.gov/pubmed?term=Crea%20F%5BAuthor%5D&cauthor=true&cauthor_uid=24519926), [Watahiki A](http://www.ncbi.nlm.nih.gov/pubmed?term=Watahiki%20A%5BAuthor%5D&cauthor=true&cauthor_uid=24519926), [Quagliata L](http://www.ncbi.nlm.nih.gov/pubmed?term=Quagliata%20L%5BAuthor%5D&cauthor=true&cauthor_uid=24519926), [Xue H](http://www.ncbi.nlm.nih.gov/pubmed?term=Xue%20H%5BAuthor%5D&cauthor=true&cauthor_uid=24519926), [Pikor L](http://www.ncbi.nlm.nih.gov/pubmed?term=Pikor%20L%5BAuthor%5D&cauthor=true&cauthor_uid=24519926), [Parolia A](http://www.ncbi.nlm.nih.gov/pubmed?term=Parolia%20A%5BAuthor%5D&cauthor=true&cauthor_uid=24519926), et al. Identification of a long non-coding RNA as a novel biomarker and potential therapeutic target for metastatic prostate cancer. [Oncotarget](http://www.ncbi.nlm.nih.gov/pubmed/?term=24519926) 2014; 5(3):764-774.
3. Livak KJ, Schmittgen TD. Analysis of relative gene expression data using real-time quantitative PCR and the 2(-Delta Delta C(T)) Method. Methods. 2001; 25(4):402-408.
4. Dobin A, Davis CA, Schlesinger F, Drenkow J, Zaleski C, Jha S, et al. STAR: ultrafast universal RNA-seq aligner. Bioinformatics. 2013; 29:15-21.
5. Anders S, Huber W. Differential expression analysis for sequence count data. Genome Biol. 2010; 11:R106.
